# Exploring Multimorbidity Through the Use of Deterministic and Probabilistic Record Linkage of Data in Five Health Facilities in Nairobi

**DOI:** 10.23889/ijpds.v9i5.2737

**Published:** 2024-09-18

**Authors:** Daniel Nderitu, Isaac Kisiangani, Nkosinathi Masilela

**Affiliations:** 1 Nairobi; 2 APHRC; 3 MRC/Wits Rural Public Health and Health Transitions Unit

## Introduction

Data collected by Health Demographic Surveillance Systems (HDSSs) at the community level can support very powerful research questions if it was linked to records at health facilities. In particular, understanding multimorbidity and building predictive models for key health outcomes requires linked data collected both in the community and health facilities. Additionally, the linked data can assist health management teams at the national, county, and local levels in making evidence-based choices.

## Problem

The inability to link population level data with records at health facilities affects the ability to use data to solve key health research questions. lack of common identifiers affects the possibility of integrating clinic records with population-based data.

## Methodology

We intend to link data from five health facilities in Nairobi County serving the population in the Nairobi Urban Health Demographic Surveillance System (NUHDSS) where longitudinal data was collected between 2001 and 2018. The longitudinal data is from a database of about 260,000 individuals. This will result in a merged dataset and will also provide an opportunity to assess the accuracy of the deterministic and probabilistic record linkage algorithm compared with other methods.

Note: We will have results by the time the conference is happening as groundwork has been put.

## Conclusion

The growing burden of multimorbidity on the African continent requires creative and effective strategies of addressing the problem through utilization of existing longitudinal data. By merging clinic records with population-based data, this initiative will promote the use of data evidence informed decision making for interventions to address multimorbidity.

